# Association of folate concentrations with clinical signs and laboratory markers of chronic enteropathy in dogs

**DOI:** 10.1111/jvim.16681

**Published:** 2023-03-14

**Authors:** Tarini V. Ullal, Stanley L. Marks, Sara N. Huebner, Sandra L. Taylor, Courtney D. Shelley

**Affiliations:** ^1^ Department of Medicine and Epidemiology School of Veterinary Medicine, University of California Davis Davis California USA; ^2^ School of Veterinary Medicine University of California Davis Davis California USA; ^3^ Division of Biostatistics, School of Medicine University of California Sacramento California USA

**Keywords:** CIBDAI, folic acid, food‐responsive enteropathy, immunosuppressant‐responsive enteropathy, inflammatory bowel disease

## Abstract

**Background:**

Serum folate is considered a biomarker of chronic enteropathy (CE) in dogs, but few studies have examined associations with markers of CE.

**Hypothesis/Objectives:**

To evaluate serum folate concentrations in dogs with and without CE and associations with sample hemolysis and selected markers of CE. We hypothesized that hypofolatemia would be more common in dogs with CE and associated with hypocobalaminemia, higher CIBDAI, and hypoalbuminemia.

**Animals:**

Six hundred seventy‐three dogs with available serum folate measurements performed at an academic veterinary hospital between January 2016 and December 2019.

**Methods:**

Medical records were retrospectively reviewed to categorize cases as CE or non‐CE and record clinical details and laboratory markers. Relationships between serum folate, cobalamin, and CE variables were assessed using chi‐square, Kruskal‐Wallis, or Spearman's correlation tests.

**Results:**

Of the 673 dogs, 99 CE were compared to 95 non‐CE. In the overall cohort, serum folate concentration did not correlate with sample hemolysis (*P* = .75). In the CE subset, serum folate and cobalamin concentrations were positively associated (rho = 0.34, FDR = 0.02). However, serum folate concentrations (median [25th, 75th percentiles]) were higher (CE: 12.1 (8.9, 16.1), non‐CE: 10.4 (7.2, 15.5); *P* = .04) and cobalamin concentrations were lower (CE: 343 (240, 597), non‐CE: 550 (329, 749); *P* = .001) in the CE vs non‐CE group. Serum folate was not associated with markers of CE, but serum cobalamin was associated with albumin (*P* = .04) and cholesterol (*P* = .03).

**Conclusions and Clinical Importance:**

Hypofolatemia is an inferior biomarker of CE compared to hypocobalaminemia.

**Abbreviations**
ARE: antibiotic‐responsive enteropathy
CE: chronic enteropathy
CIBDAI: Canine Inflammatory Bowel Disease Activity Index
FDR: false discovery rate
FRE: food‐responsive enteropathy
IRE: immunosuppressant‐responsive enteropathy
non‐CE: non‐chronic enteropathy
NRE: nonresponsive enteropathy
PLE: protein‐losing enteropathy

## 1 INTRODUCTION

Chronic enteropathy (CE) is an umbrella term used for chronic gastrointestinal diseases in dogs that have clinical signs for ≥3 weeks of hyporexia, vomiting, diarrhea, or weight loss. Chronic enteropathy is diagnosed by excluding extragastrointestinal diseases and infectious, endocrine, or neoplastic causes of gastrointestinal disease.^1^ Chronic enteropathy cases can be subclassified by the response to dietary intervention (food‐responsive enteropathy: FRE), immunosuppression (immunosuppressant‐responsive enteropathy: IRE), antimicrobials (antibiotic‐responsive enteropathy: ARE), or a lack of response to all 3 therapies (nonresponsive enteropathy: NRE).^1^, ^2^ A variety of markers exist to diagnose, monitor, and prognosticate CE in dogs. Scoring indices such as the Canine Inflammatory Bowel Disease Activity Index (CIBDAI)^3^ or Canine Chronic Enteropathy Clinical Activity Index (CCECAI)^4^ quantitatively assess the severity of clinical disease. Clinicopathologic variables that assess disease severity include serum albumin,^4^, ^5^, ^6^ cholesterol,^7^, ^8^ vitamin D,^5^, ^9^ cobalamin (vitamin B12),^4^ and folate (vitamin B9) concentrations.^10^, ^11^ Derangements in the aforementioned nutrients can indicate malabsorption and convey disease severity. Panhypoproteinemia indicates protein malabsorption suggestive of a protein‐losing enteropathy (PLE).^12^ Hypocobalaminemia and hypofolatemia indicate malabsorption and localize disease to the distal (ileum) or proximal gastrointestinal tract (duodenum and proximal jejunum) where cobalamin and folate are respectively absorbed.^11^ Hypocobalaminemia and hyperfolatemia suggest small intestinal dysbiosis because of bacterial consumption of cobalamin^13^ and overproduction of folate.^14^, ^15^ Because cobalamin and folate are instrumental in 1‐carbon metabolism, deficiencies in both affect DNA methylation and synthesis, which compromises hematopoiesis and cell development.^16^

Hypocobalaminemia occurs in 19% to 54% dogs with CE,^4^, ^17^, ^18^, ^19^, ^20^, ^21^ and is associated with markers of severe disease and poor outcomes such as hypoalbuminemia,^4^ ileal intraepithelial lymphocytes,^20^ crypt abscessation and lacteal dilation,^22^ refractory treatment response, and mortality.^4^, ^17^ Serum folate is commonly measured with serum cobalamin, but only a few studies have evaluated serum folate concentration in dogs with CE^17^, ^18^, ^19^ or its association with serum cobalamin concentration and other markers of CE.^22^, ^23^, ^24^ There is no association between hypofolatemia and intestinal crypt abscessation or lacteal dilation,^22^ nor, in dogs with inflammatory PLE, is there an association between serum folate concentration and second‐line immunosuppressant treatment or survival outcome.^24^ An inverse correlation between serum cobalamin and folate concentrations in dogs with inflammatory PLE has been attributed to small intestinal dysbiosis.^24^ Hemolysis can release folate from red blood cells and subsequently, increase plasma folate concentrations in humans,^25^ but whether this occurs in serum samples from dogs is unknown.

We hypothesized that hypofolatemia would be significantly associated with hypocobalaminemia, high CIBDAI scores, and hypoalbuminemia.

## 2 MATERIALS AND METHODS

### 2.1 Medical record review and CE vs non‐CE case definitions

A retrospective review of medical records from the University of California, Davis, Veterinary Medical Teaching Hospital between 1 January 2016 to 31 December 2019 was performed to include client‐owned dogs whose serum folate concentrations had been measured. Serum cobalamin measurements and hemolysis indices associated with the samples were recorded when available. Serum folate and cobalamin measurements were performed using a chemiluminescence assay (Immulite 2000 analyzer, Siemens Medical Solutions Diagnostics). At the diagnostic laboratory utilized in this study, the average interassay coefficient of variation for serum folate measurements was 6.1% at the low level of the reference interval and 5.2% at the high level. Intraassay coefficients of variation were 3.0% at the low level and 2.7% at the high level. For serum cobalamin measurements, the average interassay coefficient of variation was 5.5% at the low level of the reference interval and 6.8% at the high level. Intraassay coefficients of variation were 7.4% at the low level and 5.6% at the high level. If dogs were receiving cyanocobalamin or folic acid supplementation at the time of presentation to the University of California, Davis, Veterinary Medical Teaching Hospital, those cases were excluded. Dogs were also excluded from study entry if they were receiving medications such as sulfasalazine or methotrexate that could inhibit folate metabolism or absorption. If dogs had multiple serum folate measurements performed between 2016 and 2019, data from the earliest visit was analyzed. Remaining cases were classified as CE cases, non‐chronic enteropathy cases (non‐CE), or indeterminate using the following definitions. A CE case had to meet 1 of the following 2 criteria: 1. Clinical signs of gastrointestinal disease such as inappetence, weight loss, vomiting, or diarrhea for ≥3 weeks, or 2. Biochemical evidence of panhypoproteinemia (serum albumin and globulin concentrations below laboratory reference intervals) or hypoalbuminemia <3.4 g/dL with serum globulin <2.0 g/dL or serum cholesterol <200 mg/dL using University of California, Davis, Veterinary Diagnostic Laboratory reference intervals (serum albumin 3.4‐4.3 g/dL, serum globulin 1.7‐3.1 g/dL, serum cholesterol 139‐353 mg/dL). Selected cut‐offs for serum globulin and serum cholesterol were decided by the authors based on clinical experience and reports of dogs with PLE having serum globulin and cholesterol concentrations on the lower end of the established reference interval.^24^, ^26^, ^27^, ^28^ The diagnostic work‐up required for inclusion of a CE case included history, physical exam, complete blood count, serum chemistry profile, and urinalysis. Other diagnostics were variably performed, but not required for inclusion. For instance, fecal centrifugation and flotation, canine pancreatic lipase, canine trypsin‐like immunoreactivity, basal cortisol, pre‐ and postprandial bile acids, abdominal ultrasound, and endoscopic or full thickness gastrointestinal biopsies. Diagnostics were performed at the discretion of the attending clinician. Dogs receiving treatments other than folate or cobalamin supplementation for their CE were not excluded from study entry. However, if these treatments led to partial control or resolution of clinical signs at the time of serum folate measurement, these dogs were excluded.

Chronic enteropathy dogs were subcategorized as FRE, IRE, both food and immunosuppressant responsive (FRE + IRE), ARE, or NRE. When CE cases could not be categorized because of an incomplete diagnostic work‐up, inappropriate treatment protocol (strict diet trial was not followed for the appropriate duration), or lack of follow‐up, these uncategorized CE cases were excluded.

An FRE was defined as considerable improvement or resolution of clinical signs associated with gastrointestinal disease in response to ≥2 weeks of a new diet (hydrolyzed, limited ingredient, fat restricted, or high fiber). An IRE was defined as a failed diet trial and considerable improvement or resolution of clinical signs associated with gastrointestinal disease with ≥4 weeks of immunosuppressant therapy (prednisone, budesonide, cyclosporine, azathioprine, or chlorambucil or some combination of these medications). A combination FRE + IRE was defined as considerable improvement or resolution of clinical signs associated with gastrointestinal disease with combination diet trials and immunosuppressant therapies. An ARE was defined as considerable improvement or resolution of clinical signs associated with gastrointestinal disease with ≥7 days of tylosin or metronidazole after failure to improve with dietary or immunosuppressant therapy. Granulomatous colitis cases were not classified as ARE. Cases with clinical improvement in response to tylosin or metronidazole administration for ≥7 days were not classified as ARE if dietary trials or immunosuppressant trials were not attempted because FRE and IRE were not appropriately excluded per antimicrobial guidelines for chronic diarrhea.^29^ However, response to ≥7 days tylosin or metronidazole therapy was recorded separately as complete resolution of clinical signs (complete improvement), partial (incomplete resolution of clinical signs), or a complete lack of clinical response to an antibiotic trial. An NRE was defined as failure to clinically improve with diet trials, immunosuppressant therapies, and antimicrobial therapies.

Dogs were categorized non‐CE if a non‐CE disease was suspected to be the cause of clinical signs or labwork abnormalities associated with gastrointestinal disease. Examples of non‐CE diseases included renal (chronic kidney disease > Stage 2 or acute kidney injury) and hepatic disease (uncontrolled chronic hepatitis), neoplasia, pancreatitis, exocrine pancreatic insufficiency, gastrointestinal foreign body, or immune‐mediated diseases (thrombocytopenia, anemia, or polyarthritis).

Features collected from each medical record at the visit where serum folate was measured were age (in months), sex (male or female, spayed, neutered, or intact), breed, body weight (kilograms), and body condition score (BCS on a scale of 1 to 9). For CE cases, the following clinical data were also obtained: CIBDAI score, duration of clinical signs associated with gastrointestinal disease (months), diarrhea type (small, large, mixed, no diarrhea, or indeterminate), presence or absence of PLE, and whether serum cobalamin or folate concentration was being checked for the first time or rechecked as a follow‐up test or visit. Hematologic and biochemical data obtained within 1 month of serum folate measurements were recorded. This data included hematocrit (Hct), hemoglobin (Hgb), mean corpuscular volume (MCV), mean corpuscular hemoglobin concentration (MCHC), reticulocyte count, neutrophil count, eosinophil count, albumin, globulin, cholesterol, glucose, blood urea nitrogen (BUN), creatinine, total magnesium (Mg), and total calcium (Ca).

The CIBDAI score was calculated as described^3^ using clinical history provided in the medical record from the visit when serum folate concentration was measured. Diarrhea type was characterized as small bowel based on presence of normal to slightly increased defecation frequency (up to 4 times a day), normal to large volume feces, weight loss, vomiting, or a combination of these features. Large bowel diarrhea was characterized based on marked increased defecation frequency (>4 times per day), small volume feces, urgency, tenesmus, hematochezia, mucoid feces, or a combination of these features. If features of both small and large bowel diarrhea were noted, the diarrhea was characterized as mixed bowel. A PLE was defined as panhypoproteinemia based on the specific laboratory's reference intervals or hypoalbuminemia <3.4 (reference interval 3.4‐4.3 g/dL) paired with either inappropriately low serum globulin concentration <2 g/dL (reference interval 1.7‐3.4 g/dL) or inappropriately low serum cholesterol concentration <200 mg/dL (reference interval 129‐353 mg/dL) using University of California, Davis, Veterinary Diagnostic Laboratory reference intervals.

### 2.2 Statistical analysis

Statistical analysis was performed using R Statistical Computing Software Version 4.0.3. Quantitative variables were summarized as means (SD) or median [first quartile: Q1, third quartile: Q3] and categorical variable as counts and percentages. All statistical tests were 2‐sided and evaluated at a significance level of 0.05 except as noted.

#### 2.2.1 Associations between serum folate, serum cobalamin, and hemolysis index in the overall study cohort

Values of serum folate and cobalamin concentrations beyond laboratory detection limits (folate >24.0 ng/mL, cobalamin >1000 ng/L or <150 ng/L) were reported as above or below these thresholds. Analysis of these data were conducted in 2 ways. First, the values were categorized as low (<6.5 ng/mL), normal (6.5‐18.6 ng/mL), or high (>18.6 ng/mL) concentrations. Serum cobalamin readings were categorized into subgroups of markedly low (<150 ng/L), low (150‐399 ng/L), normal (400‐1000), or high (>1000 ng/L) concentrations. Second, we imputed concentrations below or above the limits of the assay (cobalamin <150 or >1000 ng/L, folate >24.0 ng/mL) as the numerical assay limits. For example, serum cobalamin concentrations <150 were imputed as 150 ng/L. Pearson's chi‐square test was used to test for differences in observed and expected frequencies between serum folate and cobalamin categories. Then, to identify which cells were contributing to a significant result, adjusted standardized residuals were calculated. Spearman rank correlation tests were performed to evaluate the linear relationships between numeric values of serum folate with serum cobalamin concentrations and hemolysis index.

#### 2.2.2 Demographic, serum folate, and serum cobalamin comparisons between CE and non‐CE groups

To compare characteristics between CE and non‐CE groups, 2‐sample *t*‐tests (age, body weight, BCS) or Wilcoxon rank sum tests (cobalamin, folate concentrations) were used for quantitative variables and Pearson's chi‐square test for sex and folate, cobalamin categories.

#### 2.2.3 Associations between serum folate, serum cobalamin, and clinical and clinicopathologic variables in the CE group

Within the CE group, to evaluate relationships between serum folate concentration, serum cobalamin concentration, and numerous clinical and clinicopathologic variables, Spearman's correlation tests were used to assess relationships between serum folate and cobalamin concentrations with quantitative variables and Kruskal‐Wallis tests were used to test for median differences in serum folate and cobalamin concentrations for categorical variables. The CE variables evaluated comprised of 4 demographic variables: age, weight, BCS, and sex; 7 disease‐related variables: CIBDAI score, duration of clinical signs associated with gastrointestinal disease, diarrhea type, CE type (FRE dogs were compared to dogs requiring immunosuppressants: IRE and FRE + IRE dogs), presence or absence of PLE, response to antibiotic trials of tylosin or metronidazole, new or recheck serum cobalamin and folate; and 15 laboratory‐derived variables: Hct (%), Hgb (g/dL), MCV (fL), MCHC (g/dL), reticulocyte count (cells/μL), neutrophil count (/μL), eosinophil count (/μL), albumin (g/dL), globulin (g/dL), cholesterol (mg/dL), glucose (mg/dL), BUN (mg/dL), Cr (mg/dL), total Mg (mg/dL), and total Ca (mg/dL). Because of the large numbers of statistical tests, false discovery rates (FDR) were calculated from raw *P*‐values to adjust for multiple testing separately for serum folate and cobalamin evaluations. False discovery rate (FDR) values <0.1 were considered significant.

## 3 RESULTS

### 3.1 Overall study cohort of dogs and subsets of CE and non‐CE cases

**FIGURE 1:**
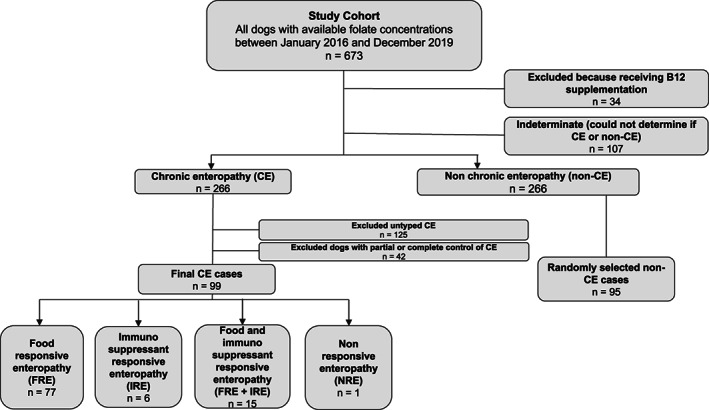
Flow diagram showing exclusion of cases for several reasons (receiving vitamin B12 supplementation, unable to categorize cases, partially or completely clinically controlled chronic enteropathy cases) yielding 99 chronic enteropathy (CE) cases and 95 randomly selected nonchronic enteropathy (non‐CE) cases.

Six hundred seventy‐three dogs had serum folate readings available for analysis during the study period of which 666 had available serum cobalamin readings. Cases receiving vitamin B12 supplementation (n = 34) were excluded leaving a remainder of 632 dogs to evaluate associations between serum folate, serum cobalamin, and hemolysis index (Table 1). A CONSORT diagram shows case inclusion and exclusion (Figure 1). After excluding cases receiving vitamin B12 supplementation, indeterminate cases (n = 107) that could not be categorized as CE or non‐CE were excluded. Of the 266 cases categorized as CE, 125 cases were excluded because they could not be typed according to treatment response and 42 were excluded because clinical signs were already partially or completely controlled at the time of folate measurement. This left 99 CE cases of which 77/99 (78%) were FRE, 6/99 (6%) IRE, 15/99 (15%) FRE + IRE, and 1/99 (1%) NRE. Of the 99 cases in the CE group, diagnostic work‐up included abdominal ultrasound in 79/99 (80%), intestinal biopsies in 29/99 (29%), basal cortisol in 52/99 (53%), specific canine pancreatic lipase in 20/99 (20%), fecal centrifugation and flotation in 17/99 (17%), and trypsin like immunoreactivity in 6/99 (6%) dogs. Of the 266 cases categorized as non‐CE, 95 were selected at random for comparison with the CE cases. Diseases diagnosed in the non‐CE group included neoplasia (n = 25) of which 9 had GI neoplasia (large cell GI lymphoma (4), gastric mass (3), small cell GI lymphoma (2)), non‐CE GI disease (n = 24) such as foreign body, pancreatitis, acute dietary indiscretion, or exocrine pancreatic insufficiency, renal disease (n = 12) of which 5 had acute and 3 had chronic disease and 4 had acute on chronic disease, hepatic or biliary disease (n = 10) such as portosystemic shunt or bacterial cholangiohepatitis, immune‐mediated disease (n = 5) such as immune‐mediated thrombocytopenia or hemolytic anemia, infectious disease (n = 4) such as leptospirosis or fungal disease, toxins or medications (n = 2), endocrine disease (n = 2) such as diabetic ketoacidosis or hypothyroidism, and other miscellaneous diseases (n = 11) such as pulmonary abscessation, inflammatory brain disease, systemic vasculitis, vaginal foreign body, bone marrow hypoplasia, biventricular congestive heart failure, and severe neuromuscular disease.

**TABLE 1:** Chi‐square test between serum cobalamin and folate subgroups in the study cohort of dogs with available serum folate and cobalamin concentrations.

		Serum folate (ng/mL)			
		Low (<6.5)	Normal (6.5‐18.6)	High (>18.6)	Total
Serum cobalamin (ng/L)	Markedly low (< 150)	7 (22.6%)	19 (61.3%)	5 (16.1%)	31 (4.91%)
	Low (150‐399)	41 (17.8%)	156 (67.5%)	34 (14.7%)	231 (36.6%)
	Normal (400‐1000)	26 (7.45%)	244 (69.9%)	79 (22.6%)	349 (55.2%)
	High (>1000)	5 (23.8%)	12 (57.1%)	4 (19.1%)	21 (3.32%)
	Total	79 (12.5%)	431 (68.2%)	122 (19.3%)	632 (100.00%)

*Note*: The study cohort comprised of 632 dogs that had available serum folate and cobalamin measurements performed between 2016 and 2019. Chi‐square test showed there were differences in the observed and expected values indicating that serum cobalamin and folate subgroups were significantly associated ($\chi^{2}$ = 22.3, *P*‐value = .001) in the study cohort.

### 3.2 Associations between serum folate, serum cobalamin, and hemolysis index in study cohort of dogs with available serum folate and cobalamin concentrations

**FIGURE 2:**
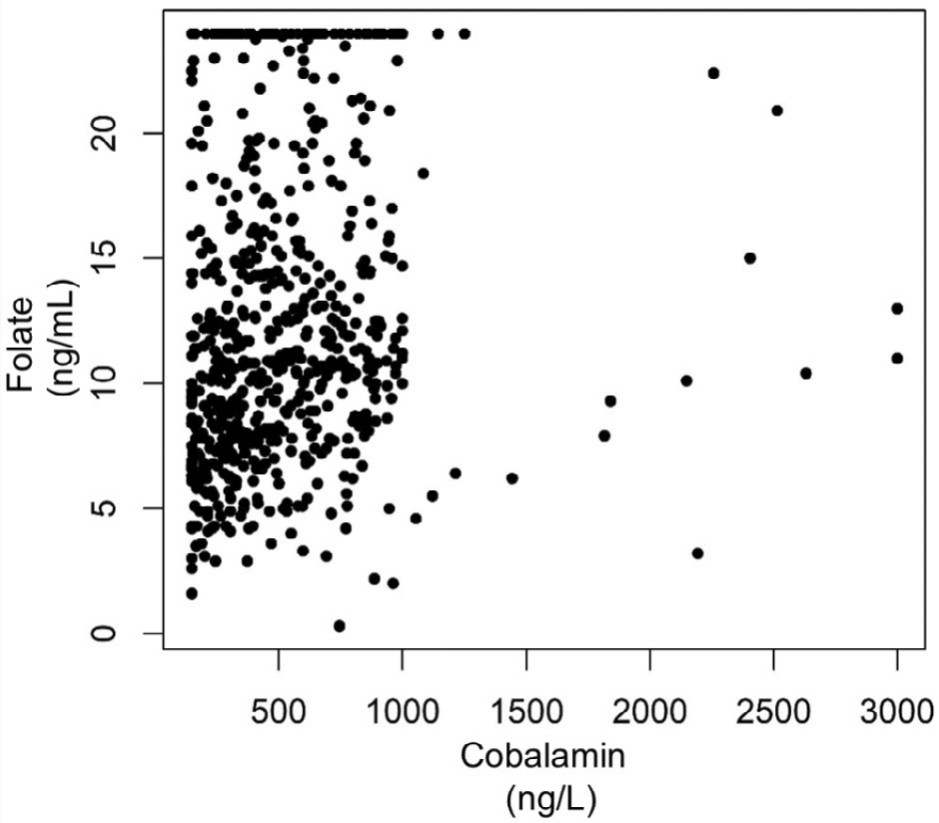
Correlation between serum folate and cobalamin concentrations in the study cohort of dogs with available serum folate and cobalamin readings. A scatter plot shows serum cobalamin concentrations on the x‐axis in ng/L and serum folate concentrations on the y‐axis in ng/mL for the study cohort of 632 dogs with available serum folate and cobalamin readings between the years 2016 and 2019. A statistically significant positive association was found between serum cobalamin and folate concentrations (rho = 0.22, *P*‐value <.001).

Serum folate concentrations were low (<6.5 ng/mL) in 81/673 (12.0%) dogs, normal (6.5‐18.6 ng/mL) in 456/673 (67.8%) dogs, and high (>18.6 ng/mL) in 136/673 (20.2%) dogs. Serum folate concentrations were >24 ng/mL in 77/673 dogs (11.4%). Of 673 dogs with serum folate measurements, 632 (93.9%) had available serum cobalamin readings and were not receiving cyanocobalamin supplementation. Of 632 dogs with available serum cobalamin readings, 31 dogs (4.9%) had markedly low (<150 ng/L), 231 dogs (36.6%) had low (150‐399 ng/L), 349 dogs (55.2%) had normal (400‐1000 ng/L) and 21 dogs (3.3%) had high concentrations (>1000 ng/L). Pearson's chi‐square test revealed significant differences in the observed and expected values indicating that serum cobalamin and folate subgroups were significantly associated ($\chi^{2}$ = 22.3, *P*‐value = .001) in the study cohort. ($\chi^{2}$ = 22.3, *P*‐value = .001; Table 1). Adjusted standardized residual analysis showed that dogs with low serum cobalamin concentrations were more likely to have low folate concentrations (Table 2). Numerical serum folate and cobalamin concentrations showed a statistically significant positive correlation (Spearman's rho = 0.22, *P* < .001; Figure 2). Serum folate concentration and hemolysis index were not correlated (Spearman's rho = −0.01, *P* = .75).

**TABLE 2:** Adjusted standardized residuals from chi‐square test between serum cobalamin and folate subgroups in study cohort of dogs with available serum folate and cobalamin concentrations.

		Serum folate (ng/mL)			
		Low (<6.5)	Normal (6.5‐18.6)	High (>18.6)	Total
Serum cobalamin (ng/L)	Markedly low (<150)	1.740	−0.847	−0.459	0.050
	Low (150‐399)	3.028	−0.272	−2.217	0.366
	Normal (400‐1000)	−4.263	1.030	2.357	0.552
	High (>1000)	1.594	−1.106	−0.030	0.033
	Total	0.125	0.682	0.193	1.000

*Note*: To identify the cells contributing to the significant chi‐square test results, cells where observed frequencies deviate significantly from expected values are highlighted. These results highlighted those cases with serum concentrations of low cobalamin and high folate, normal cobalamin and low folate, and normal cobalamin and high folate.

### 3.3 Demographics, serum folate, and serum cobalamin comparisons between CE and non‐CE groups

A total of 99 CE cases and 95 non‐CE cases were analyzed. Age in the CE group was significantly lower compared to the non‐CE group (*P* = .03). BCS was significantly higher in the CE group compared to the non‐CE group (*P* = .04). Variables of body weight and sex did not significantly differ between CE and non‐CE groups (Table 3).

**TABLE 3:** Differences in signalment, serum folate, and serum cobalamin concentrations between dogs with chronic enteropathy (CE) and non‐chronic enteropathy (non‐CE).

	CE (N = 99)	Non‐CE (N = 95)	*P*‐value
Signalment variables			
Age (months), mean (SD)	80.8 (41.2)	95.1 (51.4)	.03^a^
Body weight (kg), mean (SD)	18.9 (13.8)	17.7 (14.6)	.57^a^
BCS, mean (SD)	4.8 (1.23)	4.4 (1.51)	.04^a^
Sex			
Female, neutered	2 (2.0%)	4 (4.2%)	.48^b^
Male, neutered	42 (42.4%)	58 (49.5%)	
Female, intact	10 (10.1%)	5 (6.3%)	
Male, intact	45 (45.5%)	10 (40.0%)	
Laboratory values			
Serum folate (ng/mL), median (Q1, Q3)	12.1 (8.9, 16.1)	10.4 (7.2, 15.5)	.04^c^
Serum folate			
Low (<6.5)	8 (8.1%)	19 (20.0%)	.06^b^
Normal (6.5‐18.6)	73 (73.7%)	60 (63.2%)	
High (>18.6)	18 (18.2%)	16 (16.8%)	
Serum cobalamin (ng/L), median (Q1, Q3)	343 (240, 597)	550 (329, 749)	.001^c^
Serum cobalamin			
Markedly low (<150)	7 (7.1%)	4 (4.2%)	.01^b^
Low (150‐399)	46 (46.5%)	25 (26.3%)	
Normal (400‐1000)	44 (44.4%)	60 (63.2%)	
High (>1000)	2 (2.0%)	6 (6.3%)	

*Note*: Means and SDs for continuous variables (age, body condition score [BCS], and body weight) and counts and percentages for the categorical variable of sex are shown in the chronic enteropathy (CE) and non‐chronic enteropathy (non‐CE) groups. Age was significantly lower and BCS significantly higher in the CE group compared to the non‐CE group. Body weight and sex were not significantly different between CE and non‐CE groups. Medians and first and third quartiles (Q1 and Q3) are provided for serum cobalamin and folate concentrations in CE and non‐CE groups. Additionally, the number of cases with low, normal, or high serum folate concentrations and markedly low, low, normal, and high serum cobalamin concentrations are shown in CE and non‐CE groups. There was no significant difference in serum folate subgroups, but serum folate concentrations were significantly higher in the CE group while serum cobalamin subgroups and concentrations were significantly lower in the CE compared to the non‐CE group. Q1 = quartile 1, 25th percentile, Q3 = quartile 3, 75th percentile.

^a^ Two‐sample *t*‐test.

^b^ Chi‐square test.

^c^ Wilcoxon rank‐sum test.

Chronic enteropathy cases had a mean age of 80.8 months (SD = 41.2 months) and a body weight of 18.9 kg (SD = 13.8 kg). Most dogs were spayed or neutered (87/99, 88%). The majority were pure‐bred (74/99 [75%]) with 25/99 dogs (25%) being mixed breed. Of all dogs that presented to the academic hospital between January 2016 and December 2019, the 3 most common breeds were the Labrador retriever (3020/41051, 7.4%), chihuahua (1846/41051, 4.5%), and German shepherd (1633/41051, 4%). In the CE group, 8 dogs had subnormal serum folate (<6.5 ng/mL) concentrations and their breeds were Rhodesian ridgeback, greyhound, pitbull, Yorkshire terrier, hound mix, poodle mix, West Highland white terrier, and Shetland sheepdog. In the CE group, the 3 most common breeds with subnormal serum cobalamin concentrations (<400 ng/L) were the German shepherd (5/53, 9.4%), Labrador Retriever (3/53, 5.7%), and Yorkshire terrier (3/53, 5.7%).

Serum folate concentrations were low in 8/99 (8.1%), normal in 73/99 (73.7%), and high in 18/99 (18.2%) in the CE group. In contrast, serum folate concentrations were low (<6.5 ng/mL) in 19/95 (20.0%), normal (6.5‐18.6 ng/mL) in 60/95 (63.2%), and high (>18.6 ng/mL) in 16/95 (16.8%) in the non‐CE group. In the CE group, 7/99 (7.1%) had markedly low (<150 ng/L) and 46/99 (46.5%) had low serum cobalamin (150‐399 ng/L) concentrations in comparison to the non‐CE group in which 4/95 (4.2%) had markedly low and 25/95 (26.3%) had low serum cobalamin concentrations. Serum folate subgroups did not significantly differ (*P* = .06) between CE and non‐CE cases, but numerical serum folate concentrations were higher in CE compared to non‐CE cases (*P* = .04). Serum cobalamin subgroups (*P* = .01) and numerical serum cobalamin concentrations (*P* = .001) were significantly lower in CE compared to non‐CE cases (Table 3).

### 3.4 Associations between serum folate concentration, serum cobalamin concentration, clinical disease, and clinicopathologic markers in CE group

Descriptive statistics showing the number and percentage of cases with missing data, range, mean with SD, and median with first and third quartiles for all continuous clinical disease and clinicopathologic variables examined are listed in Table S1. Descriptive statistics showing the number and percentage of cases with missing data and proportions of cases for all categorical clinical disease variables are listed in Table S2.

In CE cases, serum folate and cobalamin concentrations were significantly positively associated with each other (rho = 0.34, FDR = 0.02). Serum folate concentration was significantly associated with eosinophil count (*P* = .01), serum glucose (*P* = .001), and BUN (*P* = .04) in raw *P*‐values, but only with serum glucose (FDR 0.01) after adjusting for false discoveries (Table S3). Serum cobalamin concentration was initially significantly positively associated with serum albumin (rho = 0.22, *P* = .04) and cholesterol (rho = 0.24, *P* = .03) concentrations on raw *P*‐values, but not after adjusting for multiple testing (FDR = 0.34) (Table S3).

Other continuous and categorical variables such as age, sex, weight, BCS, CIBDAI score, duration of clinical signs associated with gastrointestinal disease, diarrhea type, CE type, response to antibiotic trial with tylosin or metronidazole, presence of PLE, new or recheck cobalamin/folate measurement, Hct, Hgb, MCV, MCHC, reticulocyte count, neutrophil count, globulin, Cr, total Ca, and total Mg were not significantly associated with serum folate or cobalamin concentrations. Results of correlation analyses between serum folate concentration, serum cobalamin concentration, and continuous variables are shown in Table S3. Results of Kruskal‐Wallis testing for differences in median serum folate and cobalamin concentrations for categorical variables are detailed in Table S4.

## 4 DISCUSSION

This 3‐year retrospective study evaluated serum folate status in a cohort of dogs and its associations with hemolysis index, serum cobalamin status, and several clinical and clinicopathologic variables of CE. In the overall study cohort, serum folate concentration and hemolysis index were not significantly associated, suggesting that sample hemolysis did not increase serum folate concentrations. In the overall study cohort and the CE subset, serum cobalamin and folate concentrations were significantly positively associated. However, serum cobalamin concentrations were significantly lower and serum folate concentrations significantly higher in CE compared to non‐CE cases. Furthermore, serum folate concentration was not associated with any clinical or laboratory markers of CE while serum cobalamin concentration was positively associated with serum albumin and cholesterol concentrations based on raw *P*‐values. Results suggest that hypofolatemia is an inferior biomarker of CE compared to hypocobalaminemia.

In the overall study cohort, a subnormal serum cobalamin concentration <400 ng/L was far more common (41%) compared to a subnormal serum folate concentration <6.5 ng/mL (12.5%). Similarly, in the CE group, 54% of dogs had a subnormal serum cobalamin concentration and 8% had a subnormal serum folate concentration. This might have been because of the cut‐offs chosen to define subnormal serum concentrations. Subnormal serum folate was defined as concentrations below the lower limit of the reference interval (<6.5 ng/mL). Subnormal serum cobalamin was defined as concentrations <400 ng/L despite the laboratory reference interval of 271 to 875 ng/L. Ideally serum methylmalonic acid concentrations would have been measured to identify true cellular cobalamin deficiency, but these measurements could not be performed retrospectively. Therefore, a cut‐off of 400 ng/L was selected because increased methylmalonic acid concentrations indicative of cellular cobalamin deficiency tend to occur in dogs with serum cobalamin concentrations <400 ng/L.^30^ An analogous study to define cellular folate deficiency by measuring hyperhomocysteinemia has not been performed in breeds other than Greyhounds^31^ and therefore, the laboratory reference interval was used.

To subvert limitations of selected cut‐offs, categories (subnormal, normal, and elevated) as well as numerical concentrations of serum folate and cobalamin were analyzed to examine the relationship between serum folate and cobalamin status. Results showed a statistically significant positive correlation between serum folate and cobalamin concentrations in the overall study cohort and CE subset. This result was expected given findings in cross‐sectional human^32^, ^33^ and canine studies^34^ and because chronic enteropathies can diffusely affect the small bowel to cause deficiencies in both folate and cobalamin.^35^ Hypocobalaminemia can also directly result in hypofolatemia because cobalamin deficiency impairs methionine synthase, which causes 5‐methylH_4_folate trapping and subsequent folate depletion.^36^ However, when the CE group was compared to the non‐CE group, an inverse pattern was found. A higher proportion of dogs had subnormal serum cobalamin concentrations in the CE group (53/99, 54%) compared to the non‐CE group (29/95, 31%), but hypofolatemia was less common in the CE group (8/99, 8%) compared to the non‐CE group (19/95, 20%). Serum cobalamin concentrations were also significantly lower and serum folate concentrations significantly higher in CE compared to non‐CE cases.

The findings that serum folate concentrations were higher in CE cases and hypofolatemia was more common in non‐CE vs CE cases suggest that hypofolatemia is an inferior marker relative to hypocobalaminemia in differentiating CE from non‐CE disease. The factors that impact cobalamin and folate concentrations could explain this finding as intestinal malabsorption and bacterial dysbiosis both decrease cobalamin concentrations, but opposingly affect folate concentrations. Malabsorption lowers folate concentrations, but dysbiosis increases bacterial synthesis of folate to elevate or normalize folate concentrations, which can mask folate deficiency.^37^

In this study, hyperfolatemia was more common than hypofolatemia in the overall study cohort and in the CE case group. In the overall study cohort, 19% of the dogs were hyperfolatemic and 12.5% hypofolatemic. In the CE group, 18% were hyperfolatemic and 8% hypofolatemic. Aside from hypotheses of small intestinal bacterial overgrowth^38^ and excess dietary folate or supplementation, contributing factors and the clinical significance of hyperfolatemia are still unknown in dogs. In humans, hemolysis can artifactually increase folate concentrations because of release from red blood cells,^25^ but the present study did not find a correlation between hemolysis index and serum folate concentrations, suggesting that hemolysis does not increase serum folate measurements. However, clinicians might have elected not to measure serum folate on account of sample hemolysis, which would have excluded severely hemolyzed samples.

Associations of serum folate and cobalamin concentrations with clinical and clinicopathologic markers of CE were also evaluated in this study. Neither serum folate nor cobalamin concentration was associated with any of the clinical variables (CIBDAI scores, duration of clinical signs, diarrhea type, antibiotic trial response, PLE status, or food responsive vs immunosuppressant responsive CE types). This suggests that serum cobalamin and folate concentrations have limited ability to predict clinical features, disease severity, or therapeutic response to specific interventions. The inability of these B vitamins to distinguish between FRE, IRE, ARE, and other CE types is consistent with previous studies.^39^, ^40^ However, challenges in the retrospective collection of history and clinical information from medical records could have led to miscategorization of cases or missing data. Just under two‐thirds of CE dogs in this study had available CIBDAI scores and only a third of dogs received an antibiotic trial and had sufficient information to interpret treatment response. Additionally, only 99/266 (37%) CE cases had adequate medical record information to be typed.

Associations of serum folate and cobalamin concentrations with hematologic variables showed no relationship with Hct or erythrocyte indices in this study, consistent with a previous study in dogs.^23^ These findings suggest that unlike humans, dogs do not develop megaloblastic macrocytic anemia secondary to folate or cobalamin deficiencies^41^ although it is unknown how many of these dogs had true cellular deficiencies without measuring serum homocysteine and methylmalonic acid concentrations, respectively. Associations with biochemical variables showed that serum cobalamin concentration was significantly positively associated with serum albumin and cholesterol concentrations in raw *P* value form. This finding corroborated results from previous studies in dogs with CE, which have linked hypocobalaminemia, hypoalbuminemia, and poor clinical outcomes.^4^, ^17^, ^42^ However, after adjusting for false discoveries, results were no longer significant. It is possible that serum protein, cholesterol concentrations, and classification of PLE cases were affected by other factors such as hepatic dysfunction or protein‐losing nephropathies that were not definitively excluded. However, the authors attributed nonsignificant findings to small sample sizes because the same statistical analysis in a cohort of CE cases inclusive of uncategorized, untyped CE cases found statistically significant results between serum concentrations of cobalamin, folate, albumin, cholesterol, calcium, and PLE status (unpublished data). In the dataset of typed CE cases presented in this study, serum folate concentration was not associated with any previously reported clinical or laboratory markers of CE. Serum folate concentration was only significantly negatively associated with serum glucose concentration though the mechanistic explanation for this is unclear.

Main limitations of this study were because of its retrospective study design, which broached challenges in the categorization of CE cases. The diagnostic testing and treatment approach was not standardized for every dog with CE. To address this limitation, the authors used strict criteria to type CE cases by treatment response. However, assessing treatment response was a subjective process reliant upon the quality of the information provided in the medical record, reported by the pet owner and recorded by the clinician. As a result, many untyped cases were excluded, which reduced sample size and statistical power. Additionally, the inclusion of only typed CE cases might have biased the cohort to favor dogs with less severe, more treatment‐responsive disease and selected for pet owners more likely to pursue treatment and follow‐up. This was evidenced by the predominance of FRE dogs in the CE group, which is the CE type known to have the least severe and most treatable disease.^17^, ^39^ Additional evidence of this theory was found because CE dogs were significantly younger with higher BCS compared to the non‐CE group. Although these differences were found, serum folate and cobalamin concentrations were not associated with age or BCS in the CE group. Thus, differences in serum cobalamin and folate status between the CE and non‐CE groups could not be fully explained by age or BCS.

Another potential limitation was that case selection was not restricted to treatment‐naïve cases. Therefore, serum folate and cobalamin concentrations might have been affected by previous or ongoing therapies. However, exclusion of treatment naive cases would have further reduced sample size because most dogs presenting to an academic referral hospital have already received 1 or more treatments before presentation. To overcome this potential limitation, dogs already partially or completely clinically controlled at the time of serum folate measurement were excluded.

Finally, because of the large number of clinical and laboratory markers evaluated in this study, a multivariable linear regression analysis inclusive of all variables and accounting for interactions between confounding variables was not performed. Instead, exploratory univariate analyses were performed to test for associations between serum folate concentration, serum cobalamin concentration, and a number of clinical and laboratory markers individually followed by an adjustment for false discoveries. Statistical results might have differed if a multivariable linear regression approach was taken.

## CONFLICT OF INTEREST DECLARATION

Authors declare no conflict of interest.

## OFF‐LABEL ANTIMICROBIAL DECLARATION

Authors declare no off‐label use of antimicrobials.

## INSTITUTIONAL ANIMAL CARE AND USE COMMITTEE (IACUC) OR OTHER APPROVAL DECLARATION

Authors declare no IACUC or other approval was needed.

## HUMAN ETHICS APPROVAL DECLARATION

Authors declare human ethics approval was not needed for this study.

## Supporting information


**Table S1:** Descriptive statistics for continuous variables related to signalment, clinical disease, and laboratory markers in dogs with chronic enteropathy.
**Table S2:** Descriptive statistics for categorical variables related to signalment and clinical disease in dogs with chronic enteropathy.
**Table S3:** Spearman's correlation assessment of serum cobalamin or folate concentrations vs continuous variables regarding signalment, clinical disease, and laboratory markers in dogs with chronic enteropathy.
**Table S4:** Kruskal‐Wallis results evaluating associations between serum concentrations of cobalamin or folate and categorical clinical disease variables in dogs with chronic enteropathy.
