# Supplementary material for: Association of folate concentrations with clinical signs and laboratory markers of chronic enteropathy in dogs
Source: J Vet Intern Med. 2023 Mar 14;37(2):455–64. doi: 10.1111/jvim.16681 (PMC10061186; doi:10.1111/jvim.16681)
Supplement: Supplementary file 1 — Table S1: Descriptive statistics for continuous variables related to signalment, clinical disease, and laboratory markers in dogs with chronic enteropathy. Table S2: Descriptive statistics for categorical variables related to signalment and clinical disease in dogs with chronic enteropathy. Table S3: Spearman's correlation assessment of serum cobalamin or folate concentrations vs continuous variables regarding signalment, clinical disease, and laboratory markers in dogs with chronic enteropathy. Table S4: Kruskal‐Wallis results evaluating associations between serum concentrations of cobalamin or folate and categorical clinical disease variables in dogs with chronic enteropathy. [file JVIM-37-455-s001.pdf]

Supplementary Tables

Supplementary Table 1: Descriptive Statistics for Continuous Variables Related to Signalment, Clinical Disease, and Laboratory Parameters in Dogs with Chronic Enteropathy

Variable	Missingness N (%)	[Min, Max]	Mean (SD) Median (Q1, Q3)
Age (months)	0 (0%)	[3, 181]	80.8 (41.2)<br>84 (53.5, 110.0)
Weight (kilograms)	0 (0%)	[1.8, 63.8]	18.9 (13.8)<br>15.3 (6.9, 28.3)
BCS (scale of 1-9)	4 (4%)	[2, 8]	4.9 (1.2)<br>5 (4, 6)
CIBDAI Score	41 (41.4%)	[0, 11]	4.5 (2.7)<br>4.0 (2.3, 5.8)
Clinical Signs of GI disease (months)	7 (7.0%)	[0.11, 120]	27.0 (32.3)<br>10.0 (3.0, 48.0)
HCT (%)	16 (16.2%)	[14.6, 75.5]	46.3 (8.2)<br>46.7 (43.0, 50.9)
Hb (gm/dL)	16 (16.2%)	[4.5, 25.2]	15.7 (2.8)<br>16.0 (14.5, 17.5)
MCV (fl)	16 (16.2%)	[53.1, 80.7]	70.1 (3.7)<br>70.3 (68.0, 72.0)
MCHC (gm/dL)	16 (16.2%)	[30.0, 36.9]	34.0 (1.1)<br>34.1 (33.3, 34.6)
Reticulocytes (cells/µL)	79 (79.7%)	[18800, 287000]	66025 (63170)<br>41350 (22925, 94600)
Neutrophils (/µL)	16 (16.2%)	[2429, 20684]	7799 (4153)<br>6507 (4670, 10645)
Eosinophils (/µL)	17 (17.2%)	[0, 1842]	456 (424)<br>323 (159, 686)
Albumin (g/dL)	11 (11.1%)	[0.8, 5]	3.2 (0.8)<br>3.5 (2.8, 3.8)
Globulin (g/dL)	11 (11.1%)	[1.1, 5.3]	2.4 (0.7)<br>2.3 (2.0, 2.6)
Cholesterol (mg/dL)	11 (11.1%)	[66, 389]	189 (73.6)<br>177 (144, 221)
Glucose (mg/dL)	14 (14.1%)	[64, 151]	97.3 (14.7)<br>99.0 (89.0, 104.0)
BUN (mg/dL)	11 (11.1%)	[6, 87]	17.6 (10.4)<br>15.5 (12.0, 20.3)
Cr (mg/dL)	12 (12.1%)	[0.3, 2.7]	0.87 (0.36)<br>0.8 (0.7, 1.0)
Mg (mg/dL)	17 (17.2%)	[0.8, 3.5]	2.0 (0.36)<br>2.1 (1.9, 2.2)
Ca (mg/dL)	12 (12.1%)	[4.4, 11.6]	9.7 (1.2)<br>10 (9.2, 10.5)
Cobalamin (ng/L)	0 (0%)	[150, 1816]	445 (281)

			343 (240, 597)
Folate (ng/mL)	0 (0%)	[3, 24]	13.0 (5.5)<br>12.1 (8.9, 16.1)

BCS = Body Condition Score, CIBDAI = Canine Inflammatory Bowel Disease Activity Index, Hct = Hematocrit, Hgb = Hemoglobin, MCV = Mean corpuscular volume, MCHC = Mean corpuscular hemoglobin concentration, BUN = Blood urea nitrogen, Cr = Creatinine, Mg = Magnesium, Ca = Calcium, Min = Minimum, Max = Maximum, SD = standard deviation, Q1 = First Quartile, 25th percentile, Q3 = Third quartile, 75% percentile

Supplementary Table 1: Descriptive statistics for the chronic enteropathy (CE) group showing the number and percentage of cases with missing data, range, mean with standard deviation (SD), and median with first and third quartile (Q1, Q3) for all continuous clinical disease and clinicopathologic variables examined.

Supplementary Table 2: Descriptive Statistics for Categorical Variables Related to Signalment and Clinical Disease in Dogs with Chronic Enteropathy

**Variable**	**Missingness N (%)**	**Values**
Sex	0 (0%)	Female Neutered: 42 (42.4%)<br>Male Neutered: 45 (45.5%)<br>Female Intact: 2 (2.0%)<br>Male Intact: 10 (10.1%)
Diarrhea Type	0 (0%)	Large Bowel: 10 (10.1%)<br>Small Bowel: 25 (25.3%)<br>Mixed Bowel: 29 (29.3%)<br>No diarrhea: 28 (28.3%)<br>Cannot determine: 7 (7.1%)
CE Type	0 (0%)	FRE: 77 (77.8%)<br>ARE: 0 (0%)<br>IRE: 6 (6.1%)<br>FRE + IRE: 15 (15.2%)<br>NRE: 1 (1.0%)
Response to Antibiotic Trial	66 (66.7%)	Resolution of Signs: 13 (39.4%)<br>Partial Response: 13 (39.4%)<br>No Improvement: 7 (21.2%)
PLE	2 (2.0%)	Present: 31 (32.0%)<br>Absent: 66 (68.0%)
New or Recheck Cobalamin/Folate	0 (0%)	Newly measured: 92 (92.9%)<br>Recheck measurement: 7 (7.1%)

CE = chronic enteropathy, PLE = protein losing enteropathy, FRE = food responsive enteropathy, ARE = antibiotic responsive enteropathy, IRE = immunosuppressant responsive enteropathy, NRE = non-responsive enteropathy

Supplementary Table 2: Descriptive statistics for the chronic enteropathy (CE) group showing the number and percentage of cases with missing data and proportions of cases for each categorical variable are shown.

Supplementary Table 3: Spearman's Correlation Assessment of Serum Cobalamin or Folate Concentrations Versus Continuous Variables Regarding Signalment, Clinical Disease, and Laboratory Markers in Dogs with Chronic Enteropathy

	**Serum Cobalamin**			**Serum Folate**		
Variable	Spearman	Raw P	FDR	Spearman	Raw P	FDR
Age	-0.082	0.420	0.596	-0.015	0.880	0.916
Weight	-0.09	0.373	0.593	0.116	0.252	0.648
BCS	0.128	0.216	0.550	0.12	0.245	0.648
CIBDAI Score	-0.034	0.798	0.862	-0.063	0.637	0.816
Clinical Signs of GI disease	-0.01	0.928	0.928	-0.074	0.484	0.800
Hct (%)	0.177	0.108	0.524	0.061	0.582	0.816
Hb (gm/dL)	0.165	0.136	0.524	0.026	0.815	0.882
MCV (fl)	0.12	0.282	0.550	0.028	0.804	0.882
MCHC (gm/dL)	0.047	0.672	0.812	-0.043	0.697	0.824
Reticulocytes (cells/µL)	-0.12	0.285	0.550	-0.001	0.994	0.994
Neutrophil count (/µL)	0.121	0.277	0.550	0.058	0.604	0.816
Eosinophil count (/µL)	0.135	0.227	0.550	0.286	**0.009**	0.120
Albumin (g/dL)	0.222	**0.038**	0.339	-0.048	0.659	0.816
Globulin (g/dL)	0.079	0.462	0.624	0.155	0.148	0.559
Cholesterol (mg/dL)	0.237	**0.026**	0.339	0.168	0.118	0.559
Glucose (mg/dL)	-0.126	0.249	0.550	-0.368	**0.001**	**0.014**
BUN (mg/dL)	0.043	0.691	0.812	-0.221	**0.038**	0.331
Cr (mg/dL)	-0.02	0.851	0.884	-0.075	0.492	0.800
Mg (mg/dL)	0.109	0.330	0.593	-0.181	0.103	0.559
Ca (mg/dL)	0.15	0.165	0.550	0.083	0.443	0.800
Folate (ng/mL)	0.341	0.001	**0.015**	-	-	-

BCS = Body Condition Score, CIBDAI = Canine Inflammatory Bowel Disease Activity Index, GI = Gastrointestinal, Hct = Hematocrit, Hgb = Hemoglobin, MCV = Mean corpuscular volume, MCHC = Mean corpuscular hemoglobin concentration, BUN = Blood urea nitrogen, Cr = Creatinine, Mg = Magnesium, Ca = Calcium

Supplementary Table 3: This table shows Spearman rank correlation coefficients, p-values, and false discovery rate (FDR) results for associations between serum cobalamin, serum folate, and the selected clinicopathologic markers of CE (chronic enteropathy) in dogs. Significant p -values ($p < 0.05$) are bolded. Significant FDR results (FDR < 0.1) are highlighted and bolded.

Supplementary Table 4: Kruskal Wallis Results Evaluating Associations Between Serum Concentrations of Cobalamin or Folate and Categorical Clinical Disease Variables in Dogs with Chronic Enteropathy

Variable	Cobalamin KW chi-square (p-value)	Cobalamin FDR Value	Folate KW chi-square (p-value)	Folate FDR Values
Sex	2.194 (0.533)	0.685	5.309 (0.151)	0.559
Diarrhea Type	1.863 (0.761)	0.856	5.130 (0.274)	0.648
CE Type*	3.139 (0.371)	0.593	2.004 (0.572)	0.816
Response to Antibiotic Trial	1.776 (0.411)	0.596	2.180 (0.336)	0.728
PLE	2.951 (0.086)	0.524	1.681 (0.195)	0.633
New or Recheck Cobalamin/Folate	2.465 (0.116)	0.524	0.638 (0.424)	0.800

KW = Kruskal Wallis, FDR = False discovery rate, CE = Chronic enteropathy, PLE = Protein-losing enteropathy

*Due to small sample sizes of other CE types, dogs with food responsive enteropathy (FRE) were compared to dogs requiring immunosuppressant medication, which comprised of immunosuppressant responsive enteropathy (IRE) and FRE + IRE dogs.

Supplementary Table 4: This table shows Kruskal Wallis test chi-square, p-values, and false discovery rate (FDR) results for associations between serum cobalamin concentration, serum folate concentration, and categorical variables such as sex and the selected clinical disease markers of chronic enteropathy (CE) in dogs.
